# Development and Validation of a Stability-Indicating RP-HPLC Method for the Assay of Pristinamycin in Bulk and Tablet Dosage Form

**DOI:** 10.3797/scipharm.1506-01

**Published:** 2015-07-22

**Authors:** Nagasarapu Mallikarjuna Rao, Dannana Gowrisankar

**Affiliations:** 1 Research & Development, Jawaharlal Nehru Technological University, Kakinada-533003. Andhra Pradesh, India; 2 Department of Pharmaceutical Analysis and Quality Assurance, College of Pharmaceutical Sciences, Andhra University, Visakhapatnam, Andhra Pradesh, India

**Keywords:** Pristinamycin, Antibiotic, Staphylococcal, HPLC, Stability

## Abstract

Pristinamycin is an antibiotic used mainly in the treatment of *Staphylococcus* infections. The aim of this study was to develop a rapid and simple stability-indicating RP-HPLC method for the determination of pristinamycin in tablet dosage form. Pristinamycin was eluted on the ACE-5, C_18_-HL, 250 x 4.6 mm, 5 µm analytical column with a mobile phase consisting of 0.2% orthophosphoric acid and acetonitrile 63:37 v/v, pumped at 1.5 ml/min flow rate. The column was maintained at 40°C and 10 μl of the solutions were injected. UV detection was performed at 206 nm. The procedure separated pristinamycin and its potential degradation products in an overall analysis time of less than 10 min with pristinamycin eluting at about 3 min. The method was validated according to the regulatory guidelines with respect to specificity, precision, accuracy, linearity, and robustness. Forced degradation studies were also performed for pristinamycin bulk drug samples to demonstrate the stability-indicating power of the HPLC method. The % RSD of system precision and method precision was found to be 0.64 and 1.49%, respectively. The procedure provided a linear response over the concentration range 25–150 μg/ml (*r* = 0.9998). Finally, the applicability of the method was evaluated in the tablet dosage form as well as in stability samples.

## Introduction

**Fig. 1:**
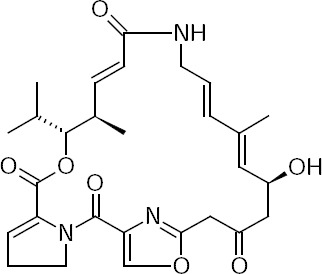
Chemical structure of pristinamycin IIA.

Methicillin-resistant *Staphylococcus aureus*, particularly if multi-resistant, is an increasing cause of nosocomial infection [1, 2]. Pristinamycin (PSM), an oral streptogramin anti-toxin, has been broadly utilized for about 50 years in the treatment of staphylococcal and streptococcal diseases. It is extracted from *Streptomyces pristinaspiralis* and has inhibitory activity against a broad range of Gram-positive bacteria. It is formed by the mixture of water-insoluble pristinamycin IA and macrocyclic lactone pristinamycin IIA. Each compound is bacteriostatic, but their association is synergic and bactericidal [3]. PSM IIA is chemically (3*R*,4*R*,5*E*,10*E*,12*E*,14*S*)-14-Hydroxy-4,12-dimethyl-3-(propan-2-yl)-8,9,14,15,24,25-hexahydro-1*H*,3*H*,22*H*-21,18-(azeno)pyrrolo[2,1-*c*][1,8,4,19]dioxadiazacyclotetracosine-1,7,16,22(4*H*,17*H*)-tetrone. Its pubchem CID number is 443309. The chemical structure of pristinamycin is shown in Figure 1.

PSM is active mainly against Gram-positive bacteria, specifically *Staphylococcus* and *Streptococcus*. However, *Neisseria spp., Mycoplasma spp., Ureaplasma spp., Chlamydia spp*. and *Haemophilus influenzae* are also susceptible [4].

To the best of the authors’ knowledge, no RP-HPLC method was found in the literature for the determination of PSM in pharmaceutical formulations. Hence, in continuing our research towards the determination of PSM, we developed a stability-indicating RP-HPLC method for the determination of PSM in bulk and tablet dosage form. The method was validated as per International Conference on Harmonization (ICH) guidelines [5].

## Materials and Methods

### Chemical and Reagents

PSM and related compounds were provided by Manus Aktteva Biopharma LLP, Gujarat, India. All HPLC-grade solvents were obtained from Merck, Mumbai, India. Potassium dihydrogen orthophosphate monohydrate, potassium hydroxide, methanol, hydrochloric acid, sodium hydroxide, and hydrogen peroxide (30%) were obtained from Merck, India. Water (18.2 MΩ.cm) was obtained using a Milli-Q system (Millipore, USA).

### HPLC Apparatus and Conditions

The Waters Alliance e2695 Separation Module (Waters Corporation, Milford, USA) equipped with a 2489 UV/Vis detector or 2998 PDA detector (for specificity and forced degradation studies) with Empower 2 software was used for the analysis. The ACE-5, C18-HL column (250×4.6 mm, 5 μm) was used. Isocratic mobile phase was composed of 0.2% orthophosphoric acid and acetonitrile in 63:37 (v/v) ratios. Water: acetonitrile 60:40 v/v was used as diluent. A flow rate of 1.5 ml/min was maintained. The eluted compounds were monitored at 206 nm. The column oven and autosampler temperatures were maintained at 40°C, respectively. An injection volume of 10 μl was used.

### Preparation of Stock Solutions

A stock solution of PSM (1.0 mg/ml) was prepared by dissolving 100 mg of PSM in a final volume of 100 ml diluent. Working standard of 100 μg/ml was prepared from the above stock solution for the related substance and assay determination, respectively.

### Preparation of Sample Solution

Twenty PSM tablets were determined, transferred to a clean and dry mortar, and ground into a fine powder. An amount of 355 mg tablet powder was then transferred to a 250 ml volumetric flask, 100 ml of diluent was added, and the flask was attached to a rotary shaker for 10 min to disperse the material completely. The mixture was then sonicated for 30 min and diluted to volume with diluent to give a solution containing 100 μg/ml. This solution was filtered through a 0.45 μm pore size Nylon 66 membrane filter.

### Method Validation

The anticipated method was validated as per ICH guidelines [5, 6].

#### Specificity (Forced Degradation Studies)

Specificity is the ability of the method to measure the analyte response in the presence of its excipients. The specificity of the developed LC method for PSM was carried out in the presence of its degradants. Stress studies were performed for PSM tablets to provide an indication of the stability-indicating property and specificity of the proposed method. Deliberate degradation was tried with the stress conditions of UV light (254 nm), acid (0.5 N HCl), base (0.5 N NaOH), and oxidation (3.0% H_2_O_2_) to evaluate the ability of the proposed method to separate PSM from its degradation product. The peak purity test was carried out for the PSM.

#### Precision

The precision of the method was verified by repeatability and intermediate precision. Repeatability was checked by injecting six individual preparations of the real PSM sample (tablets). The intermediate precision of the method was likewise assessed utilizing a distinctive examiner and performing the examination on varied days. The precision of the assay method was evaluated by carrying out six independent assays of the real sample of PSM at the 100 μg/ml level against the qualified reference standard. The intermediate precision of the assay method was evaluated by different analysts by making use of different columns and different lots of the sample.

#### Linearity

Linearity test solutions for the assay method were prepared from PSM stock solution at six concentration levels from 25 to 150% of the assay analyte concentration (25, 50, 75, 100, 125, and 150 μg/ml). The peak area versus concentration data was treated by least-squares linear regression analysis. Linearity test solutions for the method were prepared by diluting the stock solution to the required concentrations.

#### Accuracy

The assay method’s accuracy was evaluated in triplicate using the three concentration levels 50, 100, and 150 μg/ml on the real sample (tablets). Standard addition and recovery experiments were conducted on the real sample to determine the accuracy of the method. The study was carried out in triplicate using three (50, 100, and 150%) concentration levels. The percentage of recoveries for PSM was calculated.

#### Robustness

To determine the robustness of the developed method, experimental conditions were deliberately altered and the system suitability parameters were evaluated. The tailing factor for PSM was recorded. The flow rate of the mobile phase was 1.5 ml/min, and to study the effect of flow rate on the retention time, the flow was changed by ± 0.2 units from 1.5 to 1.7 and 1.3 ml/min. The effect of the column temperature on retention time was studied at 38 and 42°C.

## Results

### Specificity (Forced Degradation Studies)

**Fig. 2:**
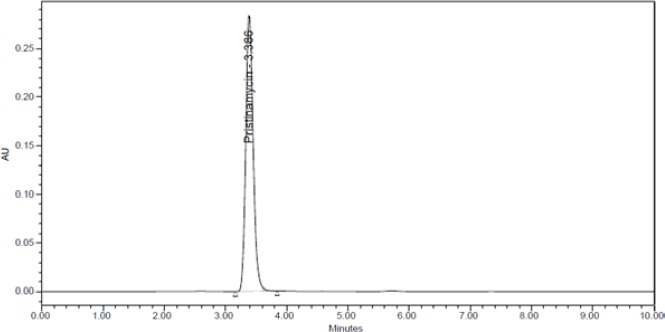
Representative HPLC chromatogram of PSM.

**Fig. 3:**
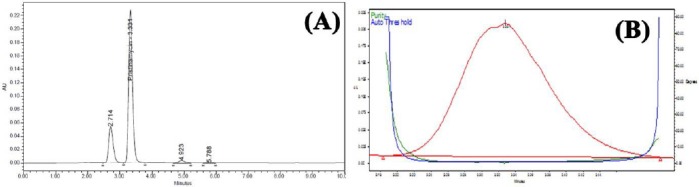
HPLC chromatogram (A) of working standard solution of PSM 100 μg/ml after acid hydrolysis (0.5 N HCl at room temperature) and (B) its peak purity plot.

**Fig. 4:**
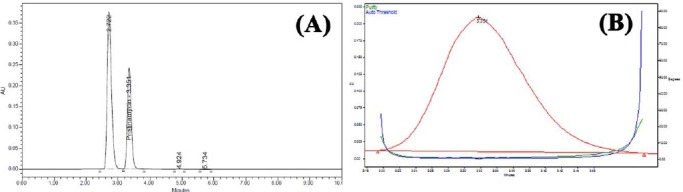
HPLC chromatogram (A) of working standard solution of PSM 100 μg/ml after alkali hydrolysis (0.5 N NaOH at room temperature) and (B) its peak purity plot.

**Fig. 5:**
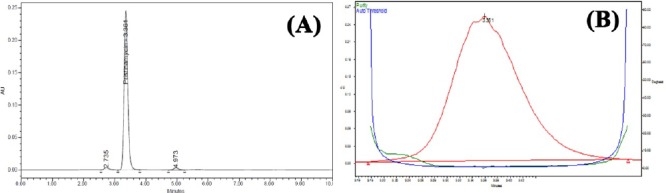
HPLC chromatogram (A) of working standard solution of PSM 100 μg/ml after UV light hydrolysis and (B) its peak purity plot.

**Fig. 6:**
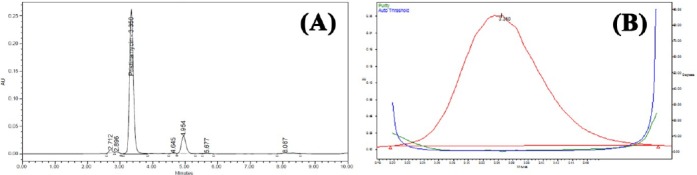
HPLC chromatogram (A) of working standard solution of PSM 100 μg/ml after oxidative degradation with 3% H_2_O_2_ hydrolysis and (B) its peak purity plot.

PSM was found to degrade significantly in acid hydrolysis and base hydrolysis, and mild degradation was observed in UV and peroxide stress conditions. Figure 2 shows the representative chromatogram of PSM and Figures 3–6 show the chromatograms of degradation studies with their purity plots. A photodiode array detector was employed to check and ensure the homogeneity and purity of the PSM peaks in all of the stressed sample solutions. Assay studies were carried out for the stress samples against PSM qualified working standard. The results are presented in Table 1. The purity and assay of PSM were unaffected by the presence of its degradation products and thus confirms the stability-indicating power of the developed method.

**Tab. 1:**
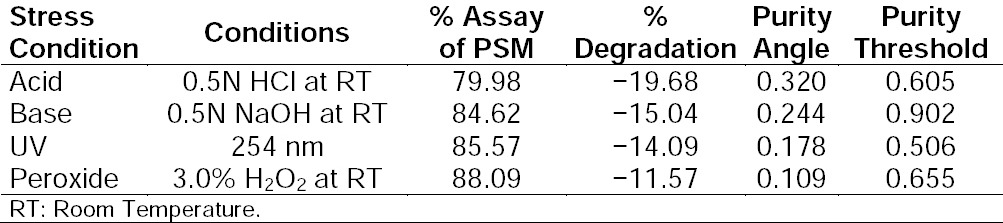
Summary of forced degradation studies

### Precision

The % RSD of the assay of PSM during the method precision study was 0.307% and 1.49% for retention time and peak area, respectively. The % RSD of the assay results obtained in the system precision study was 0.260% and 0.64% for retention time and peak area, respectively. The % RSD for the area of PSM was well within 2%, confirming good precision of the method. The % RSD values are presented in Table 2.

**Tab. 2:**
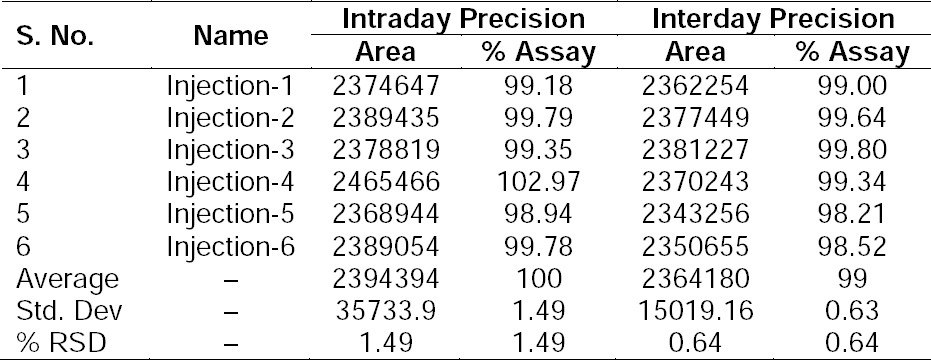
Results of Precision

### Linearity

**Fig. 7:**
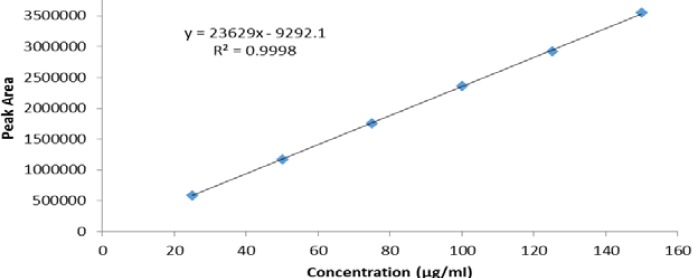
Linearity curve of PSM.

The linear calibration plot for the above method was obtained over the calibration range 25 μg/ml to 150 μg/ml (25–150% of PSM, nominal concentration 100 μg/ml) and the correlation coefficient obtained was greater than 0.998. The regression equation was y = 23629x − 9292.1. Figure 7 shows the linearity curve of PSM. The results show that an excellent correlation existed between the peak area and concentration of the analyte.

### Accuracy

Accuracy was determined by analyzing a sample of known concentration and comparing the measured value with the true value, and using the method of standard additions. Table 3 summarizes the accuracy results, expressed as percent recovery. The method showed good recovery.

**Tab. 3:**
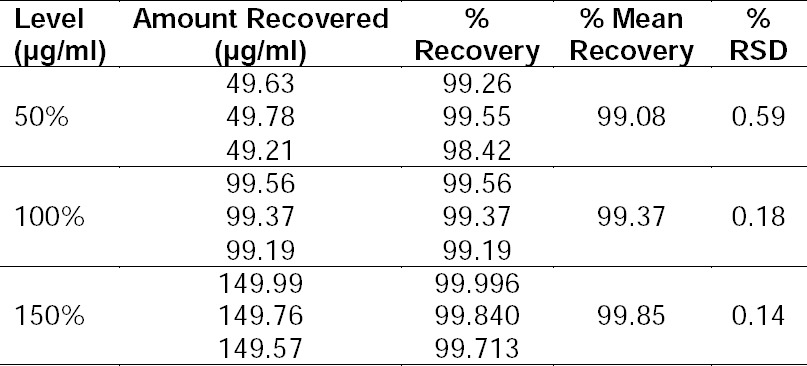
Summary of accuracy results

### Robustness

The robustness of an analytical procedure is a measure of its capacity to remain unaffected by small, but deliberate variations in method parameters, and provides an indication of its reliability during normal usage. In order to perform the robustness study of the proposed method, deliberate modifications in flow rate and column temperature were made. The results are shown in Table 4. It can be seen that for every employed condition, the chromatographic parameters are in accordance with the established value. A change in ± 0.2 units of flow rate and column temperature had no impact on chromatographic performance (Table 4). According to the data of the robustness test study, we proposed criteria for the system suitability test (tailing factors and theoretical plates number). It was used to verify that the repeatability of the system is adequate for the analysis intended.

**Tab. 4:**
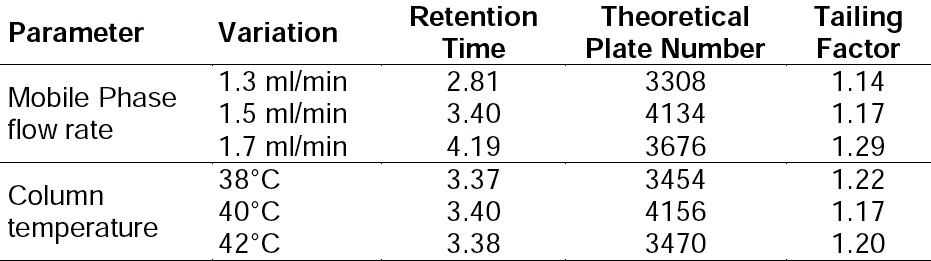
Results for the robustness study

## Discussion

Attempts were made by using different stationary phases like C_18_ and C_8_ and using a combination of buffers and organic modifiers like acetonitrile, methanol, and ethanol in the mobile phase. Selection of a suitable HPLC column was also of major concern. The ACE-5, C18-HL is high-purity, base-deactivated silica, which consists of unique C_18_ multi-alkyl bonding and exhaustive endcapping. The chromatographic separation was achieved using a mobile phase containing a mixture of 0.2% orthophosphoric acid and acetonitrile 63:37 v/v using the ACE-5, C18-HL, 250 x 4.6mm, 5µm column. A sharp peak with a good symmetry factor of PSM was noticed when the ACE-5, C18-HL column was employed. In the optimized conditions, PSM and its possible degradants were well-separated. This shows that the degradants did not have any effect on the elution of PSM. The typical retention times of PSM were about 3.5 min. The system suitability results were found to be satisfactory and the developed LC method was found to be specific for PSM.

## Conclusion

A stability-indicating RP-HPLC assay method was developed for the quantitation of pristinamycin in bulk and tablet dosage form. The developed method is specific, accurate, precise, and robust. The procedure permitted an accurate and quantitative determination of pristinamycin and its degraded products. All the degradation products formed during the forced decomposition studies were well-separated from the analyte peak and demonstrated that the developed method was specific and stability-indicating. This method can be used to carry out the analysis of pristinamycin in stability samples.
